# S-1 plus apatinib followed by salvage esophagectomy for irinotecan-refractory small cell carcinoma of the esophagus: A case report and review of the literature

**DOI:** 10.1097/MD.0000000000018892

**Published:** 2020-01-17

**Authors:** Chu Zhang, Guang-Mao Yu, Miao Zhang, Wenbin Wu, Long-Bo Gong

**Affiliations:** a Department of Thoracic Surgery, Shaoxing People's Hospital (Shaoxing Hospital, Zhejiang University School of Medicine), Shaoxing; b Department of Surgery, Xuzhou Central Hospital Affiliated to Southeast University, Xuzhou, China.

**Keywords:** apatinib, esophagectomy, S-1, salvage, serratus anterior plane block

## Abstract

**Rationale:**

Small cell carcinoma of the esophagus (SCCE) is an uncommon but lethal disease characterized by dismal prognosis. Only 10% of advanced SCCE patients survive longer than 1 year. Resection is a choice for limited-stage cases, whereas the optimal treatment regimen for primary SCCE is yet to be elucidated. To the best of our knowledge, the efficacy of S-1 plus apatinib for irinotecan-refractory SCCE has not been reported before.

**Patient concerns:**

A 61-year old, previously healthy male was admitted for dysphagia and fatigue. Endoscopic biopsy revealed a tumor in the middle third of the esophagus. Further exams including abdomen computed tomography excluded distant metastasis.

**Diagnoses:**

Primary SCCE (pT1bN1M0, IIB) was established after salvage operation.

**Interventions:**

The tumor was enlarged after 1 cycle of first-line chemotherapy using irinotecan plus cisplatin, which indicated drug resistance. Second-line oral apatinib (425 mg daily) plus S-1 (60 mg, twice daily for 4 weeks with a 2-week drug-free interval) for a month showed efficacy, as shown by decreased serum neuron-specific enolase and stable of the esophageal lesion. Thereafter, salvage minimally invasive Ivor-Lewis esophagectomy and 2-field lymph node dissection was performed, followed by oral apatinib plus S-1 at the prior dosage for 6 months. In addition, maintenance therapy using low-dose apatinib (250 mg daily) plus S-1 (40 mg, twice daily for 4 weeks with a 2-week interval) were administered for another 6 months. Then the patient was followed up irregularly at the outpatient clinic.

**Outcomes:**

The adverse events including hand-foot syndrome, hypertension, vomiting, leukopenia, impaired hepatic function, and fatigue were mainly tolerable. Forty months after the operation, he was readmitted for back pain and disseminated bone metastases appeared in magnetic resonance images. His progression-free survival could not be obtained precisely, and his overall survival was longer than 40 months up to September 2019.

**Lessons:**

S-1 plus apatinib followed by a timely esophagectomy with curative intent might be an alternative option for chemotherapy-refractory SCCE in selected patients. Better evidence is warranted.

## 1 Introduction

Primary SCCE arises from pluripotent stem cells, accounting for 0.4% to 2.8% of the esophageal malignancies.^[1]^ No treatment consensus in SCCE has been established. The therapeutic options include surgical resection, radiotherapy and chemotherapy, alone or in combination, but their prognosis remains dismal, underlining a need of new therapeutic strategies.

Apatinib is a small-molecule agent targeting vascular endothelial growth factor receptor-2 (VEGFR-2). The evidence regarding apatinib and S-1 for chemotherapy-resistance SCCE has not been reported before. Herein we present an irinotecan-refractory primary SCCE patient using S-1 plus apatinib followed by salvage radical esophagectomy, and he obtained an overall survival for more than 40 months. In addition, the literature regarding SCCE was reviewed briefly.

## 2 Case presentation

**Figure 1:**
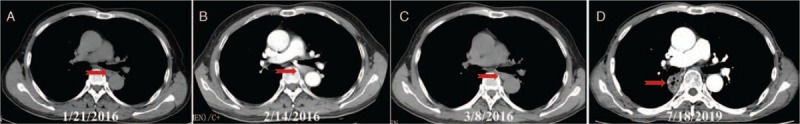
CT images of the preoperative tumor and postoperative gastric conduit (indicated by arrows). A. The middle-third esophageal wall was thickened on admission (January 21th, 2016). B. The esophageal lesion was slightly enlarged after 1 cycle of irinotecan and cisplatin. C. The tumor was stable after 1 cycle of second-line S-1 plus apatinib (1 day before surgery). D. Local recurrence was not indicated in gastric conduit about 40 months after Ivor-Lewis esophagectomy.

A 61-year-old male without smoking or drinking history was admitted on January 21th, 2016 for gradually aggravated dysphagia and fatigue, with an Eastern Cooperative Oncology Group (ECOG) score of 1. His previous history was unremarkable. Thorough physical examination failed to identify any superficial lesions, thus, laboratory, endoscopic, and radiological tests were conducted. His blood cell counts, hematocrit, hepatic and renal function, cytokeratin-19 fragment, carcinoembryonic antigen, squamous cell carcinoma, and carbohydrate antigen 125 were all in normal range, except significantly elevated serum neuron-specific enolase (NSE) of 133.0 ng/ml (normal range, 0–16.3 ng/ml). Further contrast-enhanced thoracic and abdomen computed tomography (CT) images revealed the significantly thickened middle-third esophageal wall (Fig. 1A). Esophagogastroscopy showed an irregular ulcerated lesion measuring approximately 6.0 cm × 3.0 cm, which was confirmed as primary SCCE by histopathology. Positron emission tomography was not carried out as it was not covered by his insurance. Further cranial magnetic resonance image (MRI) and bone emission computed tomography (ECT) did not indicate obviously enlarged supraclavicular lymph nodes.

The patient was afraid of surgery initially, therefore, first-line chemotherapy using irinotecan hydrochloride injection (Pfizer [Perth] Pty Limited, Australia; 65 mg/m^2^ of body surface area) combined with cisplatin (Qilu Pharmaceutical Co., Ltd., China; 80 mg/m^2^ of body surface area) was administered empirically. The efficacy was evaluated by CT images according to Response Evaluation Criteria in Solid Tumors (RECIST 1.1). However, the esophageal lesion was slightly thickened (stable disease) after 1 cycle of chemotherapy (Fig. 1B), meanwhile, the serum NSE was significantly elevated to be 217.5 ng/ml. The adverse events (AEs) included grade 2 thrombocytopenia, leukocytopenia, and grade 3 diarrhea according to National Cancer Institute Common Terminology Criteria for Adverse Events version 4.0. In addition, impaired hepatic function was indicated by elevated serum aspartate aminotransferase (AST) (250.7 U/L, normal range 1–40 U/L), and alanine aminotransferase (ALT) (189.6 U/L, normal range 1–40 U/L).

Based on these results, the patient was considered to be chemotherapy-refractory. Then he received second-line oral apatinib (425 mg, once daily) plus S-1 (60 mg, twice daily for 4 weeks with a 2-week drug-free interval, in accordance with his body surface area of 1.7 m^2^). Encouragingly, efficacy was demonstrated 1 month later, as indicated by decreased serum NSE (70.9 ng/ml) and shrinkage of the esophageal tumor (Fig. 1C).

According to the multi-disciplinary consultation and a preoperative workup, the patient was assigned to hybrid minimally invasive Ivor-Lewis esophagectomy and extended two-field lymphadenectomy on March 9th, 2016. A gastric conduit was reconstructed using end-to-side anastomosis in the upper chest.^[2]^ The operation time was 190 minutes, without obvious intraoperative blood loss. Pyloric drainage and jejunostomy for enteral nutrition were not used, except the insertion of a nasogastric tube for gastric decompression. Ultrasound-guided serratus anterior block was performed at the level of the fifth rib,^[3]^ using 0.2% bupivacaine (Marcaine, AstraZeneca, UK) at a dosage of 0.3 ml per kilogram of body weight through a 21-guage needle. Oral feeding was introduced on postoperative day (POD) 5, because no anastomotic leakage was suspected. The postoperative recovery was uneventful, and the patient was discharged on POD 9. R0 resection was achieved. The tumor in the specimen was 3.5 cm × 3 cm × 0.5 cm in size. Postoperative pathological staining revealed positive human epidermal growth factor receptor 2, VEGF, synaptophysin, chromogranin A, neuronal cell adhesion molecules (CD56), and thyroid transcriptional factor-1. The final diagnosis was G3-neuroendocrine carcinoma (small cell carcinoma) of the esophagus (pT1bN1M0, IIB) according to the 8th edition of TNM staging system for esophageal cancer.^[4]^

The patient refused postoperative mediastinal radiotherapy for personal reasons. Subsequently, oral S-1 (60 mg, twice daily of the same schedule) combined with apatinib (425 mg, once daily) was continued as adjuvant therapy for 6 months, followed by low-dose apatinib (250 mg, once daily) plus S-1 (40 mg, twice daily of the same schedule) were administered as maintenance therapy for another 6 months. Grade 2/3 AEs including hypertension, vomiting, leukopenia, impaired hepatic function (elevated serum AST and ALT), and hand-foot syndrome were controlled effectively, without grade 4 toxicity. During the adjuvant treatment, the serum NSE was maintained at 11.7 to 15.2 ng/ml (Fig. 3).

**Figure 2:**
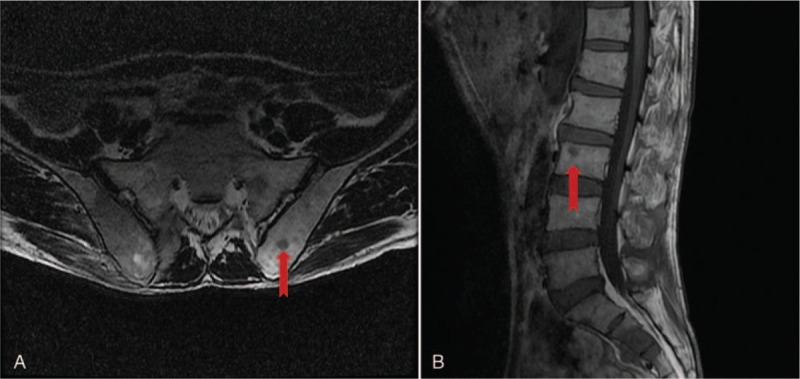
Distant metastasis was shown on his readmission at July 18th, 2019. A. The MRI images showed osteolytic lesions in the ilium. B. Another metastatic lesion was noticeable in lumbar vertebra (indicated by arrows).

The patient was readmitted to our hospital on July 18th, 2019 for gradually aggravated low back pain and loss of body weight in the previous 1 month, with compromised performance status as the ECOG score was 2. Although the CT showed normal gastric conduit, the MRI revealed disseminated osteolytic lesions in the spine and pelvis (Fig. 2), and distal metastases of SCCE was suspected. However, the patient was reluctant to be involved in a trial of anti-programmed cell death-1 (PD-1) agent, and the oral apatinib and S-1 were discontinued. Best supportive care and zoledronic acid for injection (CHIATAI TIANQING Pharmaceutical Group Co., Ltd; 4 mg every 4 weeks) was administered. The progression-free survival (PFS) of this patient could not be obtained whereas he demonstrated an overall survival (OS) for more than 40 months up to September 2019.

**Figure 3:**
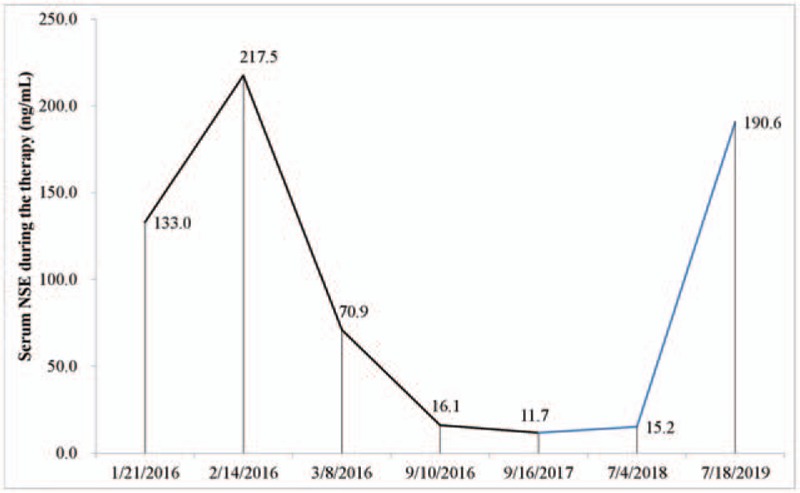
The serum NSE of this patient during the therapy.

## 3 Discussion

Primary SCCE is the most common extrapulmonary small cell carcinoma, and most SCCE patients die within 2 years of diagnosis with a median survival of 8 to 13 months.^[5]^ A systemic review of 1176 SCCE cases in China shows that, 89.7% of the tumors are located in middle and lower thoracic esophagus, with an average length of 5.4 cm (0.5–17 cm), and the median OS is 11.1 months.^[6]^ Therefore, investigation regarding the optimal therapy is urgently needed. To the best of our knowledge, this is the first report of apatinib plus S-1 followed by salvage esophagectomy in SCCE.

Apatinib monotherapy is preferred in third-line setting of advanced esophageal cancer.^[7]^ The addition of apatinib (500 mg/d) to chemotherapy could further improve the PFS of metastatic gastroesophageal junction adenocarcinoma refractory to 1 or more lines of prior chemotherapy.^[8]^ Although S-1 plus cisplatin has a high rate of AEs,^[9]^ a meta-analysis indicates that this regimen shows a favorable efficacy in gastric cancer,^[10]^ however, the findings of this review should be interpreted cautiously because of the low-quality of the included studies. The major limitations of anti-angiogenic strategy include but not limited to inevitable resistance, lacking individualized efficacy-specific biomarkers.^[11]^

The consensus of optimal treatment for SCCE is lacking to date. Stage I/IIA SCCE may be treated by surgical operation alone, especially patients with negative regional lymph nodes, and stage IIB/III disease would not benefit from operation.^[12]^ Systemic therapy could benefit the patients with advanced disease (Stage II-IV).^[13]^ A review of indicates that both esophagectomy and chemoradiation might improve the OS of the patients with limited-stage SCCE.^[14]^ Postoperative chemoradiotherapy does not improve survival in limited stage I (1–2N0M0) cases, however, chemoradiotherapy does not further improve the prognosis in completely resected limited-stage II SCCE patients as compared to chemotherapy alone.^[15–17]^ In detail, esophagectomy is the primary treatment for stage I/IIA SCCE, and neoadjuvant chemotherapy followed by esophagectomy is effective for stage ≥ IIB SCCE.^[16,18]^ Chemotherapy is associated with better survival than surgery alone or radiotherapy alone.^[19]^ Another study indicates that chemoradiotherapy is more effective than surgery and radiotherapy in limited-stage SCCE patients.^[20]^ A Surveillance, Epidemiology, and End Results (SEER) database analysis indicates that induction radiotherapy and surgery are associated with improved survival, suggesting that all SCCE patients should be considered for preoperative radiotherapy and surgery.^[21]^ On the other hand, brain metastases are uncommon in SCCE patients, and the prophylactic cranial irradiation might be unnecessary.^[22]^

A case report shows that neoadjuvant irinotecan and cisplatin (IP) followed by surgery could achieve long-term survival,^[23]^ while another case series also show that the IP regimen appears to be effective in SCCE.^[24]^ However, the presented case is refractory to irinotecan plus cisplatin but is responsive to S-1 combined with apatinib.

Furthermore, higher lymph node stage, length of tumor >3 cm,^[16]^ the depth of invasion, and chemotherapy are independent prognostic factors of SCCE,^[25]^ which recurs frequently at distant sites within 1 year after curative treatment such as definitive chemoradiotherapy.^[26]^ In addition, the recurrence time of N0/ N1 patients is significantly longer than that of the N2 patients.^[27]^ A review of 313 cases of SCCE shows that, only age (<50 years vs >50 years) and disease stage (limited stage vs extensive stage) are independent prognostic factors, and the median survival for patients with limited-stage is 17.8 months as compared to 4.9 months in those with extensive-stage disease.^[28]^ The OS of the SCCE patients with high NSE levels is worse than those with low NSE, which may be a reliable biomarker of efficacy during definitive chemoradiotherapy.^[29]^

Based on these findings, although whether surgery can prolong the survival of SCCE patients remains controversial, it seems feasible at least in part for patients with stage I/IIA SCCE. Further large studies are needed for precise selection of patients for whom surgery is indicated. Meanwhile, chemotherapy should always be part of multimodality treatment.

Additionally, SCCE has a different biological background than small cell carcinomas originating from pulmonary cells.^[5]^ Neuroendocrine neoplasm G3 of the gastrointestinal tract is defined by a proliferation index Ki67 above 20%. Definitive chemoradiotherapy with cisplatin/carboplatin and etoposide is feasible for resectable small cell neuroendocrine carcinoma of the esophagus.^[30]^ Cisplatin plus etoposide is the standard treatment regimen for advanced gastrointestinal neuroendocrine neoplasm, whereas carboplatin in combination with irinotecan could be considered as alternative first-line therapy, however, there is no standard second-line treatment.^[31]^

Lastly, next-generation sequencing enables genome-wide molecular profiling of esophageal malignancies regarding specific oncogenic pathways of tumorigenesis and progression.^[32]^ Immunotherapy treatment response is often durable, therefore, future research should focus on the combination of immunotherapy with other therapeutic modalities to increase response rate.^[33]^ A phase II interventional trial (NCT03811379) of anti-PD-1 Toripalimab (JS001) as monotherapy (240 mg, every 3 weeks until disease progress or intolerable toxicity) for SCCE patients who failed chemotherapy is ongoing, and its estimated completion date is December 30, 2021.

In summary, oral S-1 plus apatinib followed by salvage esophagectomy might be a reasonable option for selected SCCE patients. However, well-designed trials for better evidence are required to verify our findings.

## Author contributions

**Conceptualization:** Long-Bo Gong.

**Data curation:** Chu Zhang.

**Formal analysis:** Miao Zhang, Wenbin Wu.

**Funding acquisition:** Guang-Mao Yu, Long-Bo Gong.

**Methodology:** Chu Zhang.

**Resources:** Guang-Mao Yu.

**Supervision:** Miao Zhang.

**Writing – original draft:** Chu Zhang, Long-Bo Gong.

**Writing – review & editing:** Guang-Mao Yu, Wenbin Wu, Long-Bo Gong.

Long-Bo Gong orcid: 0000-0003-0644-8730.
